# The Effect of Neoadjuvant Systemic Therapy on Surgical Outcomes After Lymph Node Dissections for Stage III Melanoma; An Australian Cohort

**DOI:** 10.1245/s10434-024-15274-0

**Published:** 2024-05-18

**Authors:** Lisanne P. Zijlker, Henry Chen, Andrew J. Spillane, Maria Gonzalez, Thomas E. Pennington, Alexander M. Menzies, Serigne N. Lo, Peter Ferguson, Robert Rawson, Andrew J. Colebatch, Jonathan R. Stretch, John F. Thompson, Sydney Ch’ng, Omgo Nieweg, Kerwin F. Shannon, Georgina V. Long, Richard A. Scolyer, Robyn P. M. Saw, Alexander C. J. van Akkooi

**Affiliations:** 1 Netherlands Cancer Institute–Antoni van Leeuwenhoek (NKI-AVL), Amsterdam, The Netherlands; 2 Leiden University Medical Center, Leiden, The Netherlands; 3 Melanoma Institute Australia, The University of Sydney, Sydney, NSW Australia; 4 Faculty of Medicine and Health, The University of Sydney, Sydney, NSW Australia; 5 Royal North Shore Hospital, Sydney, NSW Australia; 6 The Mater Hospital, Sydney, NSW Australia; 7 Royal Prince Alfred Hospital, Sydney, NSW Australia; 8 NSW Health Pathology, Sydney, NSW Australia; 9 Charles Perkins Centre, The University of Sydney, Sydney, NSW Australia

**Keywords:** Melanoma, Surgery, Neoadjuvant, Systemic therapy, Complications, Textbook outcomes

## Abstract

**Background:**

Neoadjuvant systemic therapy (NAST) for patients with stage III melanoma achieves high major pathologic response rates and high recurrence-free survival rates. This study aimed to determine how NAST with targeted therapies (TTs) and immune checkpoint inhibitors (ICIs) influences surgical outcomes after lymph node dissection in terms of complications, morbidity, and textbook outcomes.

**Methods:**

Patients who underwent a lymph node dissection after either NAST in a clinical trial or upfront surgery for stage III melanoma between 2014 and 2022 were identified from an institutional research database.

**Results:**

The study included 89 NAST-treated patients and 79 upfront surgery-treated patients. The rate of postoperative complications did not differ between the NAST- and upfront surgery-treated patients (55% vs. 51%; *p* = 0.643), and steroid treatment for drug toxicity did not influence the complication rate (odds ratio [OR], 1.1; 95% confidence interval [CI], 0.4–3; *p* = 0.826). No significant differences in postoperative morbidity were observed in terms of seroma (23% vs. 11%; *p* = 0.570) or lymphedema (36% vs. 51%; *p* = 0.550). The rate of achieving a textbook outcome was comparable for the two groups (61% vs. 57%; *p* = 0.641).

**Conclusions:**

The surgical outcomes after lymph node dissections were comparable between the patients who received NAST and those who had upfront surgery, indicating that surgery can be safely performed after NAST with TT or ICI for stage III melanoma.

The introduction of adjuvant systemic therapy with immune checkpoint inhibitors (ICIs) or targeted therapies (TTs) for patients with high-risk stage III melanoma has significantly reduced recurrence rates after complete surgical resection.^1–3^ However, even after adjuvant therapy, recurrence rates remain high, with 5-year recurrence-free survival (RFS) rates of 52–55% after surgery and adjuvant treatment reported in clinical trials, and an overall survival benefit has not been observed to date.^1,2^

In pursuit of reducing recurrence rates further and ultimately improving OS, systemic therapy before surgical resection was investigated in the clinical trial setting. The rationale behind higher efficacy of the same agent given in a neoadjuvant setting is that ICI therapy induces a greater anti-tumor T cell activation, particularly in pre-existing T cell clones, when the bulk of the tumor is still present.^4–6^

Various phase 2 clinical trials have assessed different treatment regimens and demonstrated high overall and major pathologic response rates for ICI, as well as high recurrence-free survival rates, which appear superior to those achieved with adjuvant therapy.^7–11^ The phase 3 randomized NADINA trial (NCT04949113) aims to demonstrate definitively whether neoadjuvant confers a clinical benefit compared with adjuvant ICI therapy.

Surgical resection of the tumor by lymph node dissection (LND) in patients with macroscopic lymph node metastases still is the mainstay of treatment for stage III melanoma. How neoadjuvant systemic therapy (NAST) influences the surgical procedure remains uncertain.^12^ Findings have shown that ICI therapy induces tissue inflammation and fibrosis in the tumor bed or nodal basin, which could have an effect on the difficulty of the resection or the incidence of postoperative complications.^13^ Furthermore, steroid treatment for immune-related adverse events (irAEs) could theoretically affect wound-healing.

In a previous study at the Netherlands Cancer Institute (NKI), the effect of neoadjuvant systemic therapy with ICI on the surgical outcomes of LND was assessed. The findings showed no differences in terms of complications, morbidity, or textbook outcomes. However, a nonsignificantly longer duration of surgery for the neoadjuvant group was observed.^14^

In the current study we aimed to validate these findings in a larger population including not only patients who received ICI, but also those who received neoadjuvant TT.

## Methods

### Study Population

Between 2014 and 2022, all patients who underwent a LND for macroscopic stage III melanoma at Melanoma Institute Australia (MIA) were identified from the prospectively collected institutional research database. Two cohorts were defined; the first included patients who received NAST in a clinical trial before LND, and the second consisted of patients who received an upfront LND.

Data were collected from the database and supplemented by review of outpatient consultation letters. Ethnicity data were not available. The study was approved by the MIA Research Committee (MIA2022/462) and The Sydney Local Health District Ethics Review Committee (protocol nos. X15-0311 and 2019/ETH06854). All the patients had provided informed consent for inclusion of their data in the institutional database.

### Outcomes

The primary outcomes were type and incidence of postoperative complications within 90 days after the LND, graded according to the Clavien–Dindo (CD) classification (Table 1). Wound infections (including minor redness and swelling) were scored as grade 2 if treated with oral or intravenous antibiotics and grade 3 if surgical or radiologic intervention was necessary. Wound dehiscence or necrosis and hematoma were scored as grade 1 if managed conservatively and grade 3 if there was surgical intervention or negative pressure wound therapy. The secondary outcomes were postoperative morbidity (lymphedema and seroma) within 90 days and textbook outcome. Textbook outcome, a composite measure of short-term positive surgical outcomes, was defined in this study as a complete resection, no re-operation within 30 days, and no grade 2 or higher postoperative complications within 90 days.^15,16^ Lymphedema was defined as clinically apparent limb swelling treated with manual lymphatic drainage, compression therapy, or both. Seroma was defined as a fluid collection treated by aspiration or reinsertion of a drain. Also assessed was the effect of steroid treatment for irAE (scored according to the Common Terminology Criteria for Adverse Events [CTCAE] v.5) on the incidence of complications.

**Table 1:** Clavien–Dindo classification of surgical complications

Grades	Definition
1	Any deviation from the normal postoperative course without the need for pharmacologic treatment or surgical, endoscopic, or radiologic interventions
	Allowed therapeutic regimens are drugs as anti-emetics, antipyretics, analgesics, diuretics, electrolytes, and physiotherapy. This grade also includes wound infections opened at the bedside.
2	Complications requiring pharmacologic treatment with drugs other than such allowed for grade 1 complications. Blood transfusions and total parenteral nutrition are also included.
3	Complications requiring surgical, endoscopic, or radiologic intervention
3a	Intervention without patient under general anesthesia
3b	Intervention with patient under general anesthesia
4	Life-threatening complication (including CNS complications) requiring IC/ICU management
4a	Single organ dysfunction (including that requiring dialysis)
4b	Multiorgan dysfunction
5	Death of a patient

CNS, central nervous system; IC, integrated care; ICU, intensive care unit

### Treatment

All the LNDs in this study were therapeutic in nature. The study excluded patients who underwent selective lymph node resection, as in the PRADO clinical trial that excluded only the index lymph node (ILN). The patients who underwent another surgical procedure at the same time also were excluded. For groin lymph node metastases, an inguinal dissection was performed, whereas combined ilio-inguinal LND was performed when pathologically or radiologically confirmed iliac and inguinal metastases were present. Drains were left in place until drainage was 30 ml or less for two consecutive days or had been removed earlier in the event of blockage or displacement.

### Statistical Analyses

Continuous variables were summarized by medians and interquartile ranges (IQRs) and compared using the non-parametric Mann-Whitney *U* test if non-normally distributed. Normally distributed continuous variables were summarized by means and standard deviations and compared using a *t* test. Categorical variables were summarized using frequencies and percentages and compared using a Fisher’s exact test or chi square test as appropriate. Uni- and multivariable logistic regression analyses were performed to evaluate the association between NAST and predefined LND surgical outcomes. Statistical significance was set as a *p* value lower than 0.05. For statistical analysis, IBM SPSS Statistics version 27 for Windows was used.

## Results

### Patient Characteristics

The study enrolled 89 patients who received neoadjuvant systemic therapy before LND and 79 patients who received upfront LND. The patients who received NAST were younger (65 vs. 74 years; *p* < 0.001) and had a lower nodal tumor burden (N1 [74% vs. 44%]; N2 [11% vs. 29%]; N3 [15% vs. 27%]; *p* < 0.001; Table 2). The number of nodes collected per anatomic location of the dissection did not differ between the groups. Among the patients who received NAST, 29 received ICI therapy, 16 received TT, and 44 received both.

**Table 2:** Baseline characteristics of neoadjuvant-treated and upfront surgery-treated patients.

Variable	NAST (*n* = 89)* n* (%)	Upfront surgery (*n* = 79)* n* (%)	*p* value^a^
Median age: years (IQR)	74 (61–80)	65 (55.5–74)	< **0**.**001**
Gender			
Male	53 (60)	53 (67)	0.340
Female	36 (40)	26 (33)	
Primary melanoma location			0.475
Extremities	36 (40.4)	38 (48.1)	
Trunk	20 (22.5)	20 (25.3)	
Head-neck	17 (19.1)	9 (11.4)	
Unknown primary	16 (18)	12 (15.2)	
Ulceration			0.158
Absent	45 (50.6)	45 (60)	
Present	13 (14.6)	23 (29.1)	
Missing	31 (34.8)	11 (13.9)	
N stage			< **0**.**001**
pN1	66 (74.2)	35 (44.3)	
pN2	10 (11.2)	23 (29.1)	
pN3	13 (14.6)	21 (26.6)	
Dissection type			**0**.**004**
Neck	19 (21.3)	32 (40.5)	
Axillary	34 (38.2)	14 (17.7)	
Inguinal and ilioinguinal	36 (40.5)	33 (41.8)	
Median no. of resected nodes (IQR)			0.717
Neck	44 (37–68)	50 (24–61)	
Axillary	20 (16–27)	22 (17–30)	
Inguinal	9 (8–13)	12 (10–17)	
Ilioinguinal	21 (18–24)	24 (19–31)	
Median no. of involved nodes (IQR)	1 (0–2)	2 (1–3)	> **0**.**001**
Neoadjuvant therapy			
ICI	29 (32.6)		
TT	16 (18)		
ICI + TT	44 (49.4)		
Pathologic response			
Near complete response	53 (59.6)		
Partial response	14 (15.7)		
Non-response	22 (24.7)		
SLNB before LND	9 (10.1)	24 (30.4)	< **0**.**001**
RT < 90 days after LND	5 (5.6)	15 (19)	**0**.**007**

NAST, neoadjuvant systemic therapy; IQR, interquartile range; ICI, immune checkpoint inhibitor; TT, targeted therapy; SLNB, sentinel lymph node biopsy; LND, lymph node dissection; RT, radiotherapy

^a^Significant *p* values are shown in bold.

Significantly more patients in the upfront surgery group had a history of sentinel lymph node biopsy (SLNB) in the same nodal basin that received the LND (34% vs. 10%; *p* < 0.001), and more patients received adjuvant radiotherapy to the nodal basin within 90 days after surgery (19% vs. 6%; *p* = 0.007).

### Complications

**Fig. 1:**
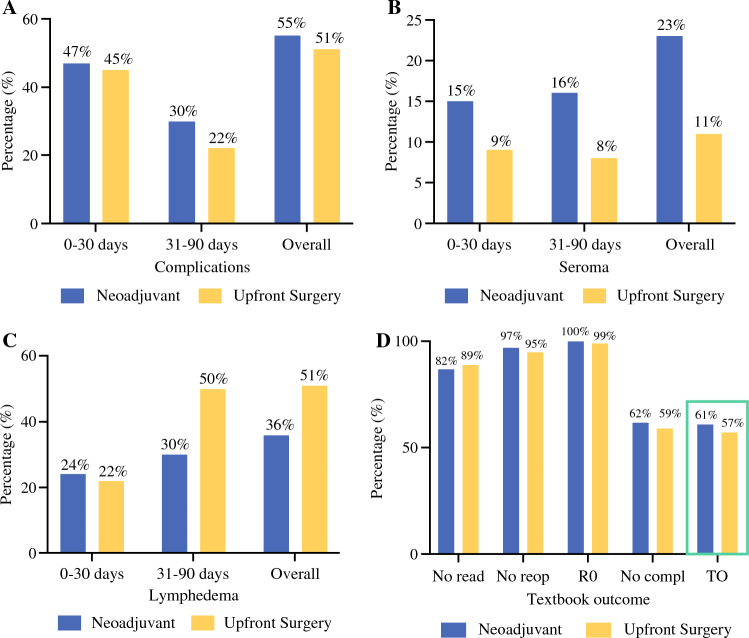
**A** Rate of complications. **B** Postoperative seroma. **C** Postoperative lymphedema. **D** Rate of patients achieving no read (no readmission), no reop (no reoperation), R0 (R0 resection), no compl (no Clavien–Dindo grade >2 complications), or TO (textbook outcome).

Within 90 days after surgery, the number of patients experiencing a surgical complication did not differ significantly between the NAST-treated group and the group that received upfront surgery (55% vs. 51%; *p* = 0.643; Fig 1A). Similarly, the rates of complications that occurred within 30 days and in the 31- to 90-day timeframe were comparable between the NAST-treated patients (47% vs. 45%; *p* = 0.877) and the upfront surgery-treated patients (30% vs. 22%; *p* = 0.211).

The most frequent complication within 30 days was a wound infection, occurring in 26% of the NAST-treated patients and 33% of the upfront surgery-treated patients (*p* = 0.395). In both groups, the wound infections were grade 2 in the majority of cases (100% vs. 92%), and only one grade 3 event occurred in the upfront-surgery group. Wound dehiscence was seen in respectively 12% and 11% of the patients (*p* = 0.847) and was mostly grade 1 (73% vs. 67%), with three grade 3 events in each group. Wound necrosis was the next most common complication, occurring in respectively 6% and 11% of the patients (*p* = 0.177), all grade 1. Neuropathy (either sensory or motor) was seen in 10% and 9% of the patients, respectively (*p* = 0.783), all grade 1. Postoperative hematoma was seen in 5% and 4% of patients, respectively (*p* = 0.821), with two grade 3 events in the NAST group. Deep venous thrombosis was seen in two patients (2%) in the NAST group.

Within the 31- to 90-day period, wound infections were the most common surgical complication, occurring in 18% of the NAST-treated patients and 17% of the upfront surgery-treated patients (*p* = 0.794). Most of the complications were grade 2, with one grade 3 and one grade 4 event occurring in the NAST group. Wound dehiscence occurred in 14% versus 5% of the patients (*p* = 0.640), with four grade 3 events in the NAST group and three grade 3 events in the upfront-surgery group. Neuropathy was observed in 3% versus 5% of the patients, respectfully (*p* = 0.584), all grade 1. Wound necrosis was seen in respectively 8% and 0% of the patients (*p* = 0.011), with one grade 3 event.

The patients who received TT had a higher overall rate of complications (75% for TT-treated, 59% for ICI-treated, and 46% for ICI+TT-treated patients), but this difference was not statistically significant (*p* = 0.113). Consistent with previous reports, there was a non-significant difference in the rate of complications within 90 days between the anatomic sites. Inguinal dissections had the highest complication rate (69%). The complication rate was 54% for ilio-inguinal dissections, 44% for axillary dissections, and 49% for neck dissections (*p* = 0.071). The patients who received TT more often had inguinal and ilioinguinal dissections (62% vs. 34%). The first operator being a consultant or surgical trainee did not make a significant difference in the rate of postoperative complications (53% vs. 69%; *p* = 0.293).

### Systemic Therapy

A TT-related adverse event graded higher than 2 was experienced by 6 patients (7%), and 18 patients (20%) experienced an immune-related adverse event graded higher than 2. A total of 21 patients (24%) received prednisone treatment before surgery, 12 of whom continued steroid treatment perioperatively. These patients were not at higher risk of experiencing postoperative complications (odds ratio [OR], 1.1; 95% confidence interval [CI], 0.4–3; *p* = 0.826). Receiving a combination of TT and ICI did not increase the risk for the development of postoperative complications (OR, 0.6; 95% CI, 0.2–1.5; *p* = 0.272).

### Morbidity

Seromas that required aspiration or reinsertion of a drain occurred in 23% of the cases in the NAST-treated group and 11% of the upfront surgery-treated patients (*p* = 0.570; Fig 1B). Similarly, the seroma rates did not differ significantly between the groups in the 30-day period (15% vs. 9%; *p* = 0.341) or in the 31- to 90-day period (16% vs. 8%; *p* = 0.151). A total of 14 patients (8%) returned to their treating physician with drain issues such as blockage or displacement.

Lymphedema that received intervention, either by treatment from a lymphedema physiotherapist or by use of compression garments, occurred in 36% of the NAST-treated patients and 51% of the upfront surgery-treated patients (*p* = 0.550; Fig 1C). Within 30 days, the rates were 24% versus 22% (*p* = 0.855), whereas at 31–90 days, the rates were 30% vs. 50% (*p* = 0.012).

The rates of seroma did not differ between the patients who received TT, ICI, or both (19% vs. 24% vs. 23%; *p* = 0.916), nor did they differ for incidence of lymphedema (38% vs. 28% vs. 41%; *p* = 0.505).

### Textbook Outcomes

The rate of patients achieving a textbook outcome did not differ significantly between the NAST-treated and upfront surgery-treated patients (61% vs. 57%; *p* = 0.641). A comparison of the separate parameters showed no differences between the groups in rate of readmission (18% vs. 11%; *p* = 0.231), reoperation within 30 days (3.4% vs. 5%; *p* = 0.584), R0 resection (100% vs. 99%; *p* = 0.287), or grade 2 or higher complications (38% vs. 41%; *p* = 0.760) (Fig 1D).

## Discussion

This study compared the surgical outcomes of LNDs between patients with stage III melanoma who received neoadjuvant systemic therapy and patients who underwent upfront LND. No difference in the rate of complications was observed (55% vs. 51%), nor did postoperative morbidity differ significantly, with a seroma incidence of 23% in the NAST group and 11% in the upfront surgery group and lymphedema in 36% and 51% of the patients, respectively. The composite measure of positive surgical outcomes, designated textbook outcomes, also was comparable between the two groups (61% vs. 57%).

The rate of grade 2 or higher complications after LND observed in this study for patients treated with NAST was in line with the previously reported complication rate in a study performed at the Netherlands Cancer Institute. Similarly, both studies showed no effect of steroid treatment for irAE’s on the rate of postoperative surgical complications. In contrast, the higher rate of complications in the upfront-surgery group at 1 to 3 months postoperatively reported in the Dutch study was not observed in the current study. Likely, this was an incidental finding, related to the lower number of patients included in the Dutch study. The current study adds to the previous study in that a higher rate of postoperative complications was not associated with neoadjuvant ICI, TT, or a combination of ICI and TT, as previously described for neoadjuvant ICI therapy only.

Although not statistically significant, a numerically higher rate of lymphedema was reported for the upfront surgery group than for the NAST-treated group. This is possibly attributable to the higher proportion of patients who had a history of SLNB in the same nodal basin, as well as to the higher rate of postoperative radiotherapy given within 90 days after surgery in the upfront-surgery group. Additionally, the patients in the upfront-surgery group had a higher nodal burden and were older than the patients in the NAST-treated group. Some physicians initiated prophylactic compression therapy and lymphedema physiotherapy. This might have led to higher rates of lymphedema reported, but would not have caused a difference in incidence between the two groups.

The incidence of seroma in this study was lower than that described in the NKI cohort, likely due to differences in drain policies. Drains at the NKI cohort were routinely removed within 1–3 days postoperatively regardless of drainage volumes, with patients presenting regularly for needle aspiration of seromas in the postoperative period. However, a considerable number of patients re-presented to the hospital with drain complications (8%) in the current study. The NAST-treated patients had a seroma rate twice as high as the upfront-surgery group. Interestingly, in the Dutch cohort, the upfront surgery group had a significantly higher rate of seroma at 1–3 months. We therefore suspect that this clinically relevant difference might have been caused by closer monitoring of the clinical trial patients.

In line with the aforementioned outcomes, the rate of patients achieving a textbook outcome was comparable between the two groups. The results of this Australian study, including neoadjuvant TT as well as ICI, thus support prior findings from a cohort of patients treated at the NKI.

Nevertheless, some limitations of this study need to be considered. First, the data were obtained retrospectively from an institutional database and from outpatient consultation letters. Complications and morbidity treated at other hospitals or by a family physician may therefore have been missed.

Furthermore, the surgeries were performed at multiple hospitals in the Sydney metropolitan area, and discharge letters were not available for all the cases in this study. Consequently, the duration of surgery and the length of admission could not always be collected, so we could not validate the previous findings in the Dutch cohort with those of this study. Additionally, although the data collection process was identical for the two groups, the clinical trial patients were likely monitored more strictly in accordance with trial protocols than the patients who received the standard of care with upfront LND. This might have led to a bias in reporting.

Finally, because the number of patients included in this cohort was relatively small, multivariable analysis of risk factors for adverse outcomes could not be performed.

## Conclusions

The postoperative outcomes of patients with stage III melanoma who received an LND after NAST or upfront LND were comparable in terms of complications, morbidity, and achievement of textbook outcomes. This study adds to the accumulating knowledge concerning the effects of NAST for both ICI and TT, and indicates that surgery after NAST can be performed safely.
